# Norwood procedure with left coronary artery reimplantation for hypoplastic left heart syndrome combined with Anomalous left coronary artery from the right pulmonary artery: a case report

**DOI:** 10.1186/s44215-026-00252-7

**Published:** 2026-03-23

**Authors:** Yuya Yamazaki, Junichi Koizumi, Tatsunori Tsuji, Daiki Saitoh, Naoya Sakoda, Azuma Tabayashi, Kazuki Yakuwa, Naoki Masaki, Hirofumi Saiki, Hajime Kin

**Affiliations:** 1 Department of Cardiovascular Surgery, Iwate Medical University, 2-1-1 Idaidori, Yahaba-cho, Shiwa-gun, Iwate, 028-3695 Japan; 2 Department of Pediatrics, Iwate Medical University, Iwate, Japan

**Keywords:** Hypoplastic left heart syndrome, Anomalous left coronary artery from the pulmonary artery, Norwood procedure, Reimplantation

## Abstract

**Background:**

Anomalous origin of the left coronary artery from the pulmonary artery is a rare congenital heart defect associated with high mortality during infancy if not surgically corrected. Its occurrence combined with hypoplastic left heart syndrome is highly uncommon, presenting considerable diagnostic and technical challenges. Identifying such an anomaly before surgery can be challenging, particularly in patients with complex cardiac anatomy. Intraoperative recognition requires immediate adjustments of the surgical approach, especially regarding myocardial protection and coronary artery reconstruction.

**Case Presentation:**

A female infant was delivered at 39 weeks of gestation with a birth weight of 3038 g. She was prenatally diagnosed with a variant of hypoplastic left heart syndrome, which was characterized by mitral and aortic stenosis, double-outlet right ventricle, and moderate tricuspid regurgitation. At birth, she underwent bilateral pulmonary artery banding to manage heart failure symptoms. Preoperative imaging did not provide sufficient visualization of the coronary arteries. At 15 days of age, she underwent the Norwood procedure with right ventricle-to-pulmonary artery shunt and tricuspid valve repair. During surgery, it was observed that the left coronary artery originated from the right pulmonary artery. To maintain coronary perfusion, a cannula was inserted into the main pulmonary artery, connecting it to a cardiopulmonary bypass circuit. Subsequently, the left coronary artery was reimplanted into the neoaorta to preserve alignment and minimize tension. The postoperative course included extracorporeal membrane oxygenation for 3 days because of right ventricular dysfunction, with sternal closure on postoperative day 13. At 7 months, she underwent a bidirectional cavopulmonary shunt. At 2 years of age, she continues to be monitored on heart failure medication and exhibits a stable clinical status.

**Conclusions:**

In patients with complex congenital heart disease, a thorough assessment of the coronary artery anatomy is essential for surgical planning. In cases of unfeasible preoperative diagnosis, prompt intraoperative management must ensure myocardial protection and long-term coronary artery function. This case demonstrates that careful intraoperative decision-making can lead to successful outcomes, even in cases of unexpected anatomical findings.

**Supplementary Information:**

The online version contains supplementary material available at 10.1186/s44215-026-00252-7.

## Background

Anomalous left coronary artery from the pulmonary artery (ALCAPA) is a rare congenital cardiac anomaly with a high mortality rate, particularly in infants, with over 90% of cases not surviving the first year of life without surgical intervention [1]. The coexistence of ALCAPA and hypoplastic left heart syndrome (HLHS) is rare; only a few cases have been reported worldwide [2]. Timely ALCAPA diagnosis is challenging, especially when associated with complex congenital heart defects, such as HLHS, which often delay detection. Intraoperative diagnosis necessitates significant changes in interventions such as cardiopulmonary bypass (CPB) perfusion, myocardial protection, and coronary and aortic arch reconstruction, increasing mortality and morbidity. Therefore, early diagnosis is critical for improving surgical outcomes.

## Case Presentation

**Fig. 1:**
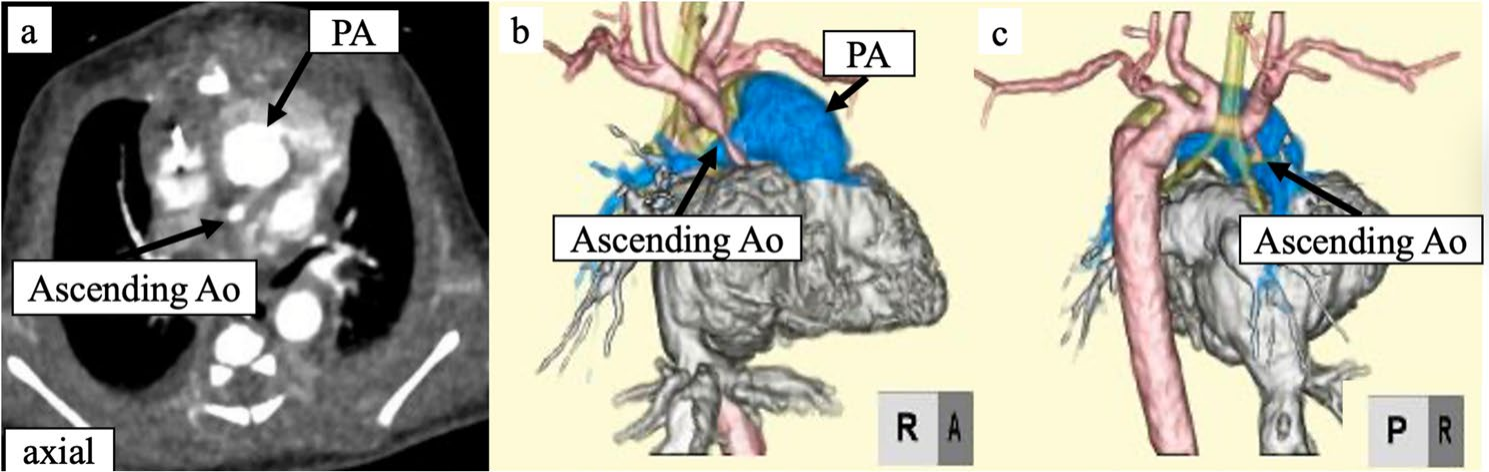
Preoperative computed tomography findings.** a** Contrast-enhanced axial scan. **b **and** c** Preoperative three-dimensional reconstruction images did not reveal a left coronary artery originating from the pulmonary artery. Ao, aorta; PA, pulmonary artery

A 28-year-old pregnant woman underwent fetal echocardiography at 35 weeks’ gestation, which raised suspicion of an HLHS variant, and she was subsequently referred to our institution. A girl delivered at 39 weeks’ gestation weighing 3038 g, with Apgar scores of 8 and 8 at 1 and 5 min, respectively. Postnatal echocardiography revealed mitral stenosis, aortic stenosis with significant left ventricular hypoplasia, and double-outlet right ventricle (RV), consistent with an HLHS variant, and moderate tricuspid regurgitation. The aortic valve measured 1.8 mm in diameter, suggesting a hypoplastic ascending aorta. Immediately after birth, the patient underwent bilateral pulmonary artery (PA) banding to manage heart failure symptoms; however, ALCAPA was not diagnosed at that time. Further evaluation using contrast-enhanced computed tomography failed to visualize the coronary arteries properly (Fig. 1), and ALCAPA was retrospectively detected during the Norwood procedure.

**Fig. 2:**
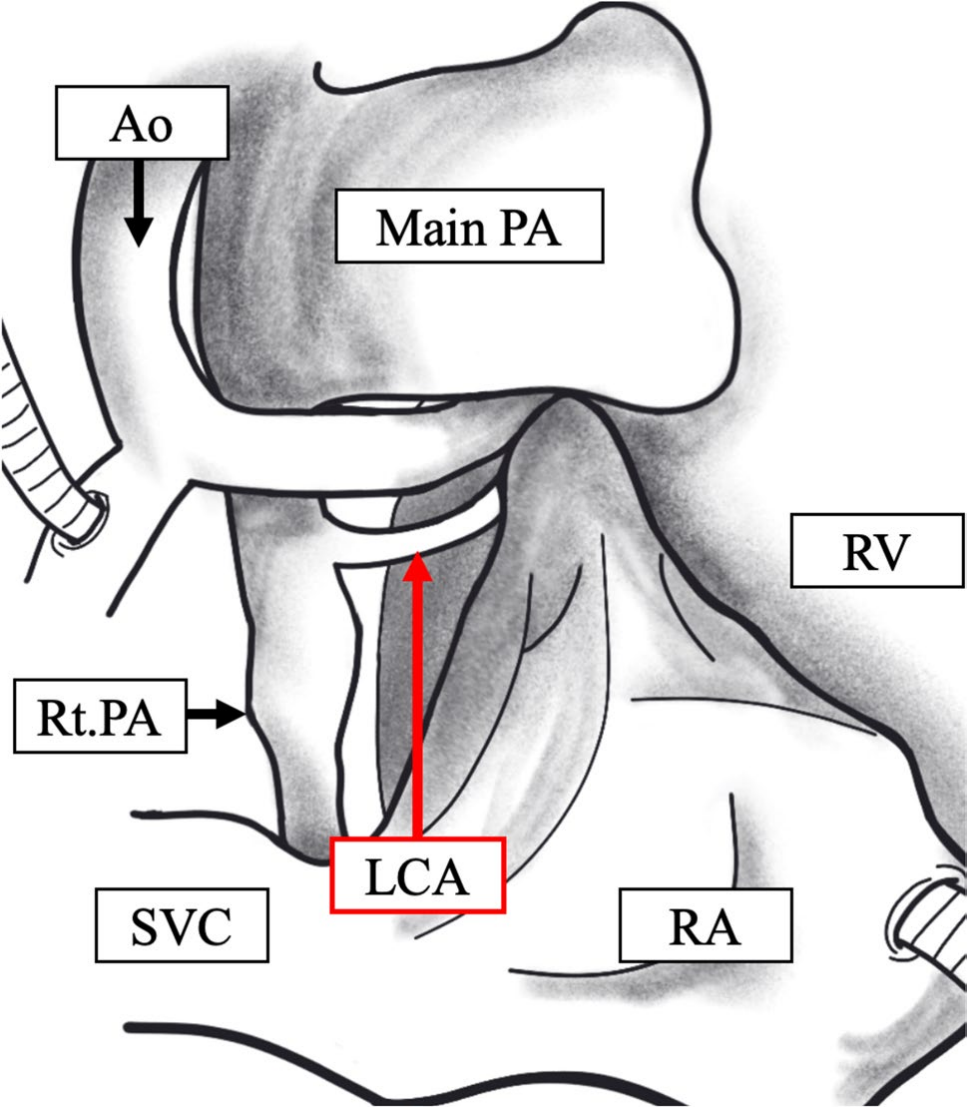
Operative findings. The image shows the left coronary artery originating from the right pulmonary artery. Ao, aorta; PA, pulmonary artery; *LCA*, left coronary artery; Rt, right

**Fig. 3:**
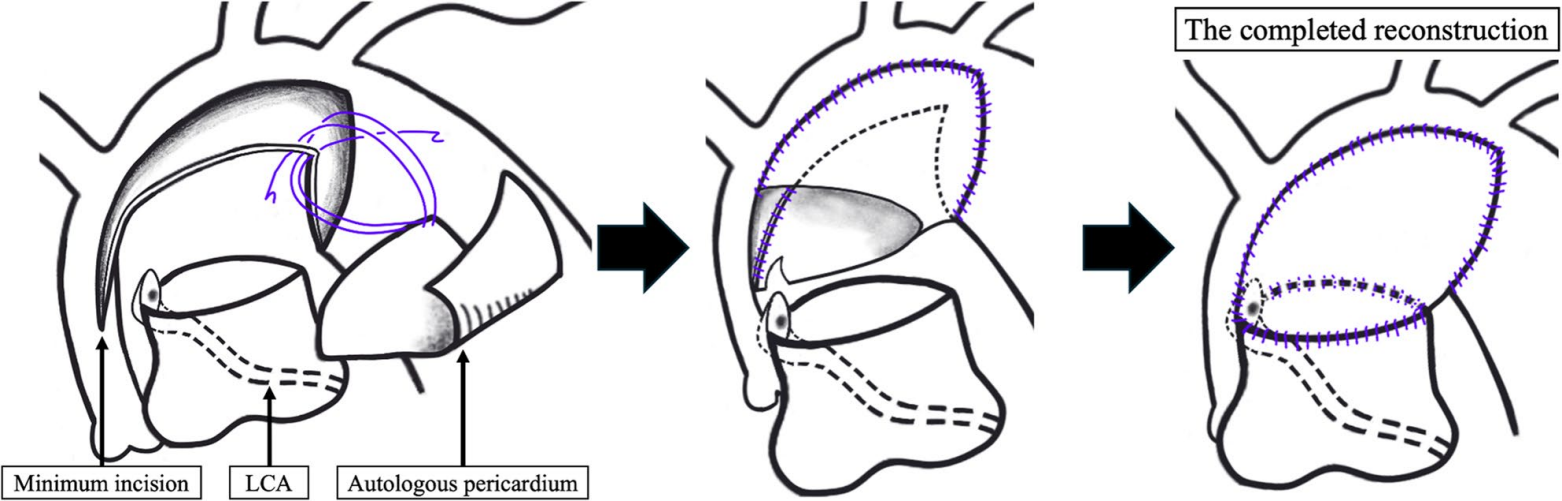
Schematic drawing of the procedure. We minimized the incision into the ascending aorta and performed anastomosis of the pulmonary artery in a more cranial position to avoid left coronary artery (LCA) blood-flow impairment

At 15 days of age, the patient underwent the Norwood procedure with an RV-PA shunt, left coronary artery (LCA) reimplantation to the neoaorta, and tricuspid annuloplasty. CPB was established via innominate artery and descending-aortic perfusion with bicaval drainage, and the patent ductus arteriosus was divided. During the dissection and debanding of the bilateral PAs, the LCA was found to originate from the right PA (RPA), confirming the ALCAPA diagnosis (Fig. 2). Consequently, we introduced a 20G cannula into the main PA (MPA) and connected it to the side arm of the CPB circuit, ensuring LCA perfusion while clamping both distal PAs to avert pulmonary steal. Atrial septal defect creation and tricuspid valve annuloplasty were performed on a beating heart. Cardiac arrest was achieved by administering cardioplegia to both the directly incised ascending aorta and the cannula connected to the MPA, with concomitant retrograde administration. Moreover, the MPA was transected just below its bifurcation, and the LCA cuff was harvested from the RPA. Following implantation of the lower half of the LCA cuff into the incised lateral wall of the MPA, the aortic arch was augmented with tanned autologous pericardium. Meticulous reconstruction avoided any tension or distortion of the LCA(Fig. 3). The distal PA defect was repaired with fresh autologous pericardium. Finally, the RV-PA conduit was anastomosed at both ends using the dunk technique.

**Fig. 4:**
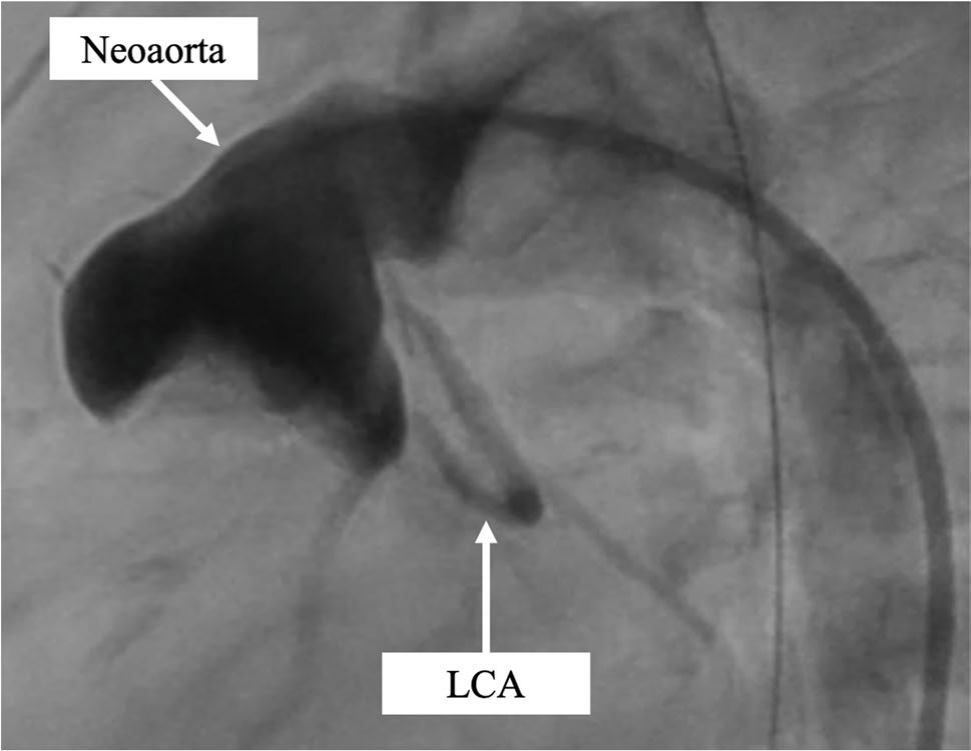
Postoperative angiography findings. The image shows good flow through the implanted left coronary artery (LCA) and no residual coarctation of the aorta

The patient was successfully weaned from 3 days of extracorporeal membrane oxygenation support for RV dysfunction and underwent delayed sternal closure on postoperative day 13 with recovered RV motion. Cardiac catheterization at 6 months of age revealed no residual coarctation of the aorta, good flow through the implanted LCA, mild RV dysfunction with a 34% RV ejection fraction, mild tricuspid regurgitation, and no PA stenosis (Fig. 4). The patient subsequently underwent a bidirectional Glenn procedure at 7 months of age. She is currently 2 years old, continues taking heart failure medication, and is regularly followed up at the outpatient clinic.

### Discussion and Conclusions

The combination of ALCAPA and HLHS is exceedingly rare; additionally, owing to its complexity, it is often diagnosed only during surgery [3]. Nathan et al. reported six patients with HLHS or its variants and ALCAPA among 552 patients undergoing stage 1 palliation [4]. Among them, ALCAPA was diagnosed preoperatively in only one; of the remaining five, only a single patient with ALPACA identified intraoperatively survived.

When ALCAPA is detected intraoperatively, two priorities arise: (1) prompt myocardial perfusion and cardioplegic protection via the MPA and (2) precise implantation of the LCA together with the small ascending aorta. In this case, the LCA was initially intended to be reimplanted close to its original position; however, this location overlapped with the standard incision site on the MPA, which is typically used for side-to-side anastomosis between the ascending aorta and the proximal MPA. Therefore, a more proximal incision extending to the sinotubular junction of the ascending aorta was avoided, and the anastomosis between the ascending aorta and the MPA was performed in a more cranial position to prevent LCA distortion and subsequent impairment of coronary blood flow. Minimizing distortion of the LCA during reimplantation is critical for ensuring long-term coronary artery patency and preventing ischemic complications.

When ALCAPA is diagnosed preoperatively, an alternative approach, described by Konuma et al. and Riggs et al. [2], becomes possible. As a first stage, they performed bilateral PA banding and patent ductus arteriosus stenting soon after birth, followed by a comprehensive second stage 4–5 months later that combined the Norwood and Glenn procedures without reimplantation of the LCA, leaving the proximal RPA and LCA to the MPA. The RPA was divided at the distal LCA takeoff and anastomosed with the left PA or superior vena cava. This technique is challenging to perform during the neonatal Norwood procedure because of the small peripheral PA.

In conclusion, the Norwood procedure with LCA reimplantation was successfully performed for HLHS combined with ALCAPA. The incision into the ascending aorta was minimized, and the MPA was anastomosed in a more cranial position to prevent impairment of LCA blood flow. Preoperative coronary imaging, particularly in patients with complex congenital heart disease, is essential to ensure timely diagnosis and optimal surgical planning.

## Supplementary Information

Supplementary Material 1.

## Data Availability

Not applicable.

## Abbreviations

ALCAPA: anomalous left coronary artery from the pulmonary artery
HLHS: hypoplastic left heart syndrome
PA: pulmonary artery
MPA: main pulmonary artery
RPA: right pulmonary artery
RV: right ventricle
LCA: left coronary artery
